# A Review of Continuum Mechanics for Mechanical Deformation of Lipid Membranes

**DOI:** 10.3390/membranes13050493

**Published:** 2023-05-03

**Authors:** Jichul Kim

**Affiliations:** INTEGRITY Co., Ltd., 9, Gangnamseo-ro, Giheung-gu, Yongin-si 16977, Gyeonggi-do, Republic of Korea; jichul0kim@gmail.com

**Keywords:** lipid membranes, continuum mechanics, curvature elasticity, surface tension

## Abstract

Mechanical deformation of lipid membranes plays important roles in various cellular tasks. Curvature deformation and lateral stretching are two major energy contributions to the mechanical deformation of lipid membranes. In this paper, continuum theories for these two major membrane deformation events were reviewed. Theories based on curvature elasticity and lateral surface tension were introduced. Numerical methods as well as biological applications of the theories were discussed.

## 1. Introduction

Lipid membranes are two-dimensional fluid sheets composed of phospholipids in a bilayer structure [1]. The lipid membrane serves as a platform for cells to communicate with the outside world. Mechanical deformation of lipid membranes plays many important roles in cellular tasks, such as signal transduction, exo- and endocytosis, and cellular remodeling. Continuum theories for lipid membranes were widely used to explain these biological processes [2,3,4,5,6,7,8,9,10,11,12,13,14,15,16]. By using elastic moduli, such as bending modulus, the continuum membrane models provided analytic solutions for long wavelength deformation shapes of membranes as well as changes in forces, energies, and the number of lipids in continuous membranes.

In this paper, theoretical backgrounds for continuum theories based on curvature elasticity [17,18] and lateral membrane surface tension [19,20] were reviewed. Elastic energy functionals and their components were discussed. In addition, previously introduced numerical models, in particular finite element methods (FEMs), to use the continuum theories were discussed. Finally, previous researches on the application of the continuum theories for cellular biological and physiological problems were introduced.

Together with other reviews on the topic of continuum mechanics of lipid membranes [21,22,23,24], this paper may provide in-depth insights into physical interpretations for the membrane curvature energy; a combined picture for the curvature deformation and lateral stretching of membranes; how the bending modulus and the surface tension of membranes can be defined; how the fluidity of lipid membranes was handled in numerical models; and how the continuum theories had been validated experimentally.

## 2. Curvature Elasticity Theory

The curvature elasticity theory of lipid membranes was proposed by Canham [17] and Helfrich [18]. The theory considers energies stored in a continuous membrane due to mean and Gaussian curvatures.

(1) $$\Psi_{\mathrm{curvature}}=\int_{\Omega }^{}\left(2{\kappa H}^{2}+\;\bar{\kappa }K\right)\mathrm{dA}$$

In the energy functional $\Psi_{\mathrm{curvature}}$, H is the mean curvature and can have both positive and negative values. The mean curvature energy density can be defined as a quadratic form of the mean curvature H. κ is the bending modulus. K is the Gaussian curvature. $\bar{\kappa }$ is the Gaussian curvature (or saddle-splay) modulus. dA is area elements, and Ω indicates the domain of integration.

**Figure 1:**
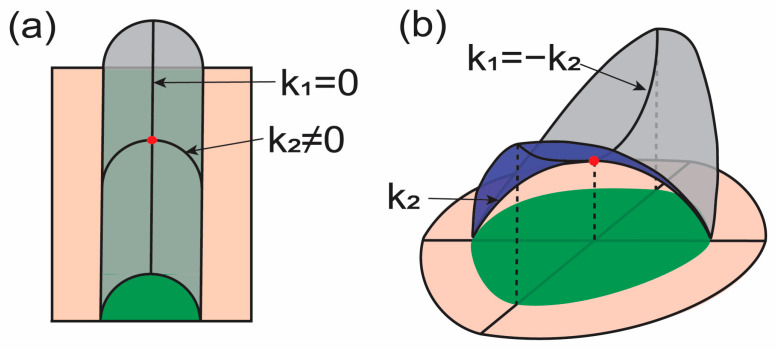
Illustrations for the curvature deformation of membranes. Surfaces shown with magenta and gray have the same surface area. $k_{1}$ and $k_{2}$ indicate principal curvatures at the marked point (red). Green regions illustrate the projected area. (**a**) The mean curvature at the red point can be changed without changing the Gaussian curvature with the given condition. (**b**) The Gaussian curvature at the red point can be changed without changing the mean curvature with the given condition.

Equation (1) can be also expressed as follows

(2) $$\Psi_{\mathrm{curvature}}=\int_{\Omega }^{}\left(\kappa \frac{k_{1}^{2}+2k_{1}k_{2}+k_{2}^{2}}{2}+{\bar{\kappa }k}_{1}k_{2}\right)\mathrm{dA},$$

where $k_{1}$ and $k_{2}$ are two principal curvatures. From Equation (2), we can interpret the physical meaning of each term. First, when one principal curvature is zero, i.e., $k_{1}=0$, and the other is nonzero, i.e., $k_{2}\neq 0$, the Gaussian curvature is zero (see Figure 1a). Therefore, the energy in this case is expressed only with respect to $k_{2}^{2}$, i.e., the square of one principal curvature. Therefore, the mean curvature energy term may consider how the surface is curved into two principal directions. $k_{1}k_{2}$ in the mean curvature energy term is identical to the Gaussian curvature expression. Now, let us consider $k_{1}=-k_{2}$ (Figure 1b). In this case, the mean curvature is zero while the Gaussian curvature is a nonzero negative value. These two principal curvatures with the same magnitude in opposite directions represent saddle-like membrane surfaces. The energy is increased when the saddle-like surface is stretched by holding $k_{1}=-k_{2}$. Therefore, the Gaussian curvature energy term may consider membrane lateral stretching.

In the three-dimensional space, the mean curvature H at a point of surfaces described by coordinates X, Y, and the height function Z can be calculated from Equation (3) [22,25].

(3) $$H=\frac{\left(1+Z_{,Y}^{2}\right)Z_{,\mathrm{XX}}-2Z_{,X}Z_{,Y}Z_{,\mathrm{XY}}+\left(1+Z_{,X}^{2}\right)Z_{,\mathrm{YY}}}{2\sqrt{{\left(1+Z_{,X}^{2}+Z_{,Y}^{2}\right)}^{3}}}.$$

In Equation (3), a comma was used to indicate differentiation, e.g., $\frac{\partial Z}{\partial X}=Z_{,X}$, $\frac{\partial^{2}Z}{\partial X^{2}}=Z_{,\mathrm{XX}}$. The Gaussian curvature can be written as follows [22,25].

(4) $$K=\frac{Z_{,\mathrm{XX}}Z_{,\mathrm{YY}}-Z_{,\mathrm{XY}}^{2}}{{\left(1+Z_{,X}^{2}+Z_{,Y}^{2}\right)}^{2}}.$$

For the rotational axisymmetric configuration, Equations (5) and (6) can be derived from Equations (3) and (4), respectively, by defining $Z=h\left(r\right)=h\left(\sqrt{X^{2}+Y^{2}}\right)$ where $r=\sqrt{X^{2}+Y^{2}}$ [26].

(5) $$H=0.5\left(\frac{h_{,\mathrm{rr}}}{{\sqrt{1+h_{,r}^{2}}}^{3}}+\frac{h_{,r}}{r\sqrt{1+h_{,r}^{2}}}\right),$$

(6) $$K=\frac{h_{,\mathrm{rr}}h_{,r}}{r{\left(1+h_{,r}^{2}\right)}^{2}},$$

where h indicates height with respect to radius r. Parametric derivatives can be used to express curvatures in Equations (3)–(6) with respect to parametric domains. This allows us to describe overhang geometries [26]. In the rotational axisymmetric case, parametric derivatives are $h_{,r}=h_{,s}/r_{,s}$ and $h_{,\mathrm{rr}}=h_{,\mathrm{ss}}/r_{,s}^{2}-h_{,s}r_{,\mathrm{ss}}/r_{,s}^{3}$, where s represents the parametric coordinate [26].

## 3. Lateral Strain Energy

### 3.1. Surface Tension versus Lateral Strain Relations

Lateral stretching of lipid membranes results in the expansion of their surface areas. Experiments were performed by Evans and his colleagues to investigate surface tension vs. lateral strain relations as well as to measure bending κ and apparent area stretching $K_{\mathrm{app}}$ moduli of lipid membranes [19,20]. Pressurization of giant vesicles by using micropipettes was performed. The equilibrium of forces provided surface tension σ of lipid membranes with the applied pressure [19,20].

(7) $$\sigma =\frac{\Delta {\mathrm{Pr}}_{\mathrm{micropipette}}}{2\left(1-\frac{r_{\mathrm{micropipette}}}{R_{\mathrm{vesicle}}}\right)}.$$

In Equation (7), $r_{\mathrm{micropipette}}$ is the radius of the micropipette, ΔP is the applied pressure to the vesicle, and $R_{\mathrm{vesicle}}$ is the radius of the vesicle [19,20]. The area expansion was determined from projected surface areas of the vesicle membrane. The observed surface tension vs. lateral strain relation demonstrated two physically distinctive responses in lateral stretching of the membrane [19,20]. In refs. [13,16,26], constitutive equations were defined for the surface tension as follows by using the results of refs. [19,20].

(8) $$\sigma =\sigma_{0}\exp\left(c_{1}\alpha \right)\;\mathrm{for}\;\alpha \leq \alpha_{\mathrm{crossover}},$$

(9) $$\sigma =K_{\mathrm{app}}\left(\alpha -\alpha_{\mathrm{cut}\text{-}\mathrm{off}}\right)\;\mathrm{for}\;\alpha >\alpha_{\mathrm{crossover}},$$

where

(10) $$c_{1}=8\pi \kappa /\left(k_{B}T\right).$$

**Figure 2:**
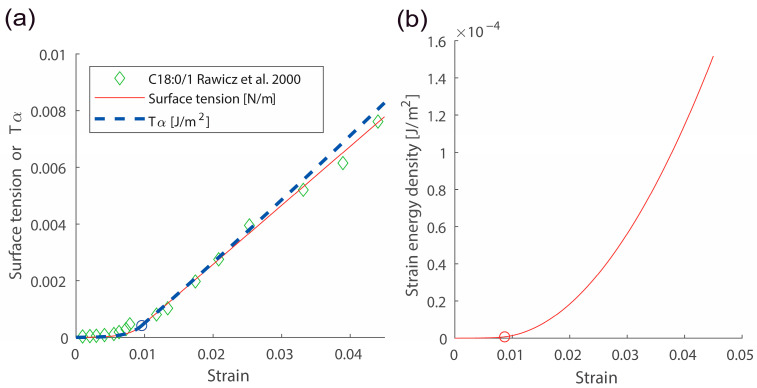
Responses due to membrane lateral stretching. (**a**) The surface tension σ vs. strain α and ${Τ}_{\alpha }$ vs. strain α responses. (**b**) The strain energy density U vs. strain α response. $\kappa =0.9\times 10^{-19}\;J$, $\sigma_{0}=\exp\left(-5.7\right)\times 10^{-3}\;N/m$, $K_{\mathrm{app}}=208\times 10^{-3}\;N/m$ were used. Red and blue circles indicate the crossover strain. Data points shown with green diamonds were obtained from ref. [20].

**Figure 3:**
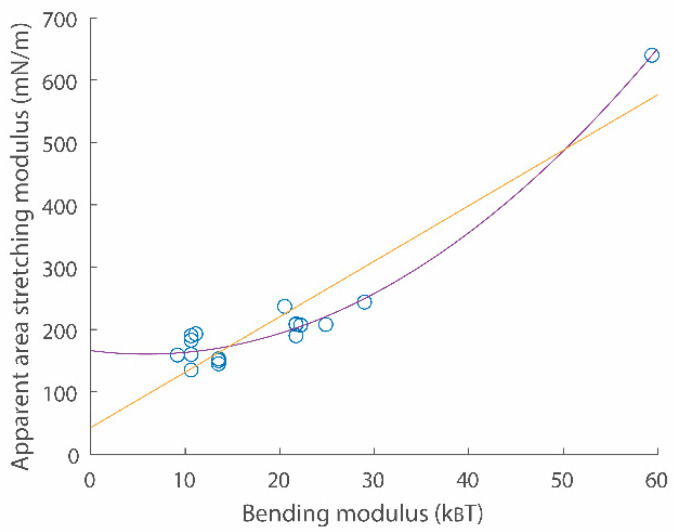
A scatter plot for apparent area stretching modulus $K_{\mathrm{app}}$ vs. bending modulus κ relations. Data points were obtained from refs. [19,20]. Second order (purple) and linear (yellow) fitting functions are $\frac{K_{\mathrm{app}}}{10^{-3}}=0.1675{\left(\frac{\kappa }{k_{B}T}\right)}^{2}-1.972\frac{\kappa }{k_{B}T}+166.1$ and $\frac{K_{\mathrm{app}}}{10^{-3}}=8.899\frac{\kappa }{k_{B}T}+42.55$, respectively.

Here, α is the membrane lateral strain. $\alpha_{\mathrm{crossover}}$ and $\alpha_{\mathrm{cut}\text{-}\mathrm{off}}$ are crossover and cut-off strains, respectively. Continuity conditions for two Equations (8) and (9) give values for $\alpha_{\mathrm{crossover}}$ and $\alpha_{\mathrm{cut}\text{-}\mathrm{off}}$. $\sigma_{0}$ is surface tension with the zero strain. $k_{B}$ is the Boltzmann constant and T is temperature. Experimental data directly demonstrated that the surface tension vs. strain relation in semi-log scales is linear with a slope $c_{1}$ in the low-strain regime [19,20]. Data also directly showed the linear surface tension vs. strain relation with a slope $K_{\mathrm{app}}$ in the high-strain regime [19,20]. An example for the surface tension vs. strain relation is shown in Figure 2a (see comparison between green data points and the red curve). A scatter plot for two important membrane parameters κ and $K_{\mathrm{app}}$ is shown in Figure 3.

In the low-strain regime, i.e., $\alpha \leq \alpha_{\mathrm{crossover}}$, stretching of entropic, i.e., non-deterministic, undulations is mainly responsible for the generation of tension (membrane thickness changes or stretching of intermolecular distances might be negligible in this regime). Theoretical investigations had been conducted to understand underlying mechanics in the low-strain regime. In refs. [21,22,27], the ratio between the excessive area due to undulations and the projected area was derived by using membrane energies expressed up to quadratic order, a square area of membranes with undulations expressed in the form of waves, and the equipartition theorem. The resulting equation was as follows.

(11) $$\frac{A_{m}-L^{2}}{L^{2}}=\frac{k_{B}T}{8\pi \kappa }\ln\left(\frac{\frac{{\kappa \pi }^{2}}{l^{2}}+\tau }{\frac{{\kappa \pi }^{2}}{L^{2}}+\tau }\right),$$

where $A_{m}$ is the total material area in the square region defined by side length L. l is the minimum length scale. τ is the chemical energy per area. By using Equation (11), strain $\alpha_{m}$ in stretching the membrane in the square region can be defined as follows [21].

(12) $$\alpha_{m}\equiv \frac{{\left.A_{m}\right|}_{\tau =0}-{\left.A_{m}\right|}_{\tau }}{L^{2}}\approx \frac{k_{B}T}{8\pi \kappa }\ln\left(1+\frac{L^{2}\tau }{{\kappa \pi }^{2}}\right)\;\mathrm{for}\;\tau \ll \frac{{\kappa \pi }^{2}}{l^{2}}.$$

${\left.A_{m}\right|}_{\tau =0}$ and ${\left.A_{m}\right|}_{\tau }$ are $A_{m}$ when τ=0 and τ≠0, respectively. In this formulation, the possibility that the bilayer thickness (or intermolecular distances) can be changed was not ruled out.

In the high-strain regime, i.e., $\alpha >\alpha_{\mathrm{crossover}}$, bilayer thickness changes or the direct expansion of intermolecular distances might be mainly responsible for the expansion of the area. The bending modulus in this regime can be smaller than the value for the low-strain regime. According to an elasticity theory, the bending modulus is proportional to the cubic of the bilayer thickness [17,28].

According to a review by Morris and Homann [29], surface tension values measured from living cell membranes can range from 0.003 mN/m to 0.04 mN/m. Adherent cells in a resting configuration are tensed, i.e., stretched in general, therefore, surface tension of membranes with the zero strain, i.e., $\sigma_{0}$, in this case, is smaller than the observed values. In ref. [16], $\sigma_{0}=\exp\left(-10\right)\;\mathrm{mN}/m$ was used to generate σ=0.0612 mN/m for resting cells on elastic substrates. This $\sigma_{0}$ value is within an order of magnitude (smaller by a factor of 2.9948) with respect to the value calculated from ${\kappa \pi }^{2}/A_{\mathrm{reservoir}}$ (see Equation (12)), where $A_{\mathrm{reservoir}}$ is the area of lipid reservoirs [16].

Mechanical properties of lipid membranes might be also estimated from the extraction of membrane tethers by using rheological methods, such as atomic force microscopy [30,31]. According to a theoretical investigation that modeled liquid membranes, the bending modulus κ of membranes and the radius of tethers satisfy a relation in Equation (13) [32].

(13) $$\frac{\kappa }{R_{\mathrm{tether}}}=\frac{f_{\mathrm{tether}\;}}{2\pi },$$

where $R_{\mathrm{tether}}$ and $f_{\mathrm{tether}}$ are the radius and pulling force of membrane tethers, respectively.

### 3.2. Lateral Strain Energy

From Equations (8) and (9), a strain energy $\Psi_{\mathrm{lateral}}$ stored in the membrane can be formulated as follows [16,26].

(14) $$\Psi_{\mathrm{lateral}}=\int_{\Omega }^{}U\left(\alpha \right)\mathrm{dA},$$

where

(15) $$U\left(\alpha \right)=\int_{0}^{\alpha }\sigma \left(\bar{\alpha }\right)d\bar{\alpha }=\frac{\sigma_{0}}{c_{1}}\exp\left(c_{1}\alpha \right)-\frac{\sigma_{0}}{c_{1}}\;\mathrm{for}\;\;\;\alpha \leq \alpha_{\mathrm{crossover}},$$

(16) $$U\left(\alpha \right)=\int_{0}^{\alpha }\sigma \left(\bar{\alpha }\right)d\bar{\alpha }=\frac{K_{\mathrm{app}}}{2}\alpha^{2}-K_{\mathrm{app}}\alpha_{\mathrm{cut}\text{-}\mathrm{off}\;}\alpha +c_{2}\;\mathrm{for}\;\;\;\alpha >\alpha_{\mathrm{crossover}},$$

(17) $$c_{2}=\frac{\sigma_{0}}{c_{1}}\exp\left(c_{1}\alpha_{\mathrm{crossover}}\right)-\frac{\sigma_{0}}{c_{1}}-\left(\frac{K_{\mathrm{app}}}{2}\alpha_{\mathrm{crossover}}^{2}-K_{\mathrm{app}}\alpha_{\mathrm{cut}\text{-}\mathrm{off}}{\;\alpha }_{\mathrm{crossover}}\right).$$

The lateral strain energy density U(α) in Equations (15) and (16) were derived by integrating the surface tension vs. membrane strain relation. See Figure 2b for an example of strain energy density U vs. strain α relation using Equations (15) and (16).

## 4. The Superposition of Curvature and Lateral Strain Energies

### 4.1. An Energy Functional

Two main modes in mechanical deformation of lipid membranes are curvature generation and lateral area stretching. For this reason, an energy function where these two energy contributions are linearly combined can be considered. The superposition of $\Psi_{\mathrm{curvature}}$ in Equation (1) and $\Psi_{\mathrm{lateral}}$ in Equation (14) were made as in Equation (18) [16,26]. In this case, the total number of lipids in the domain Ω is fixed.

(18) $$\Psi_{\mathrm{total}}=\Psi_{\mathrm{curvature}}+\Psi_{\mathrm{lateral}}\;=\int_{\Omega }^{}\left(2{\kappa H}^{2}+\;\bar{\kappa }\;K\right)\mathrm{dA}+\int_{\Omega }^{}U\left(\alpha \right)\mathrm{dA}.$$

In Equation (18), the long wavelength membrane curvature was considered in the term $\Psi_{\mathrm{curvature}}$ and the short wavelength curvature was considered in the term $\Psi_{\mathrm{lateral}}$.

**Figure 4:**
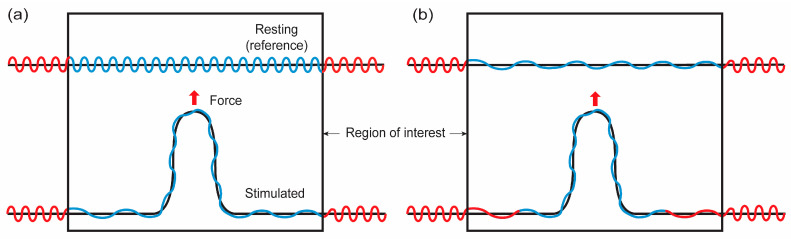
Illustrations for possible membrane configurations when membranes are point loaded. Red and blue lines represent membranes located outside and inside of the region of interest (ROI), respectively, in the resting configuration. Black lines represent the projected area of membranes with undulations (blue and red). (**a**) Closed lipid reservoir. The membrane located outside of ROI does not flow into ROI. The level of surface undulation in ROI is decreased in mechanical stretching. (**b**) Open lipid reservoir. The membrane located outside of ROI can flow into ROI.

As demonstrated in Figure 4a and discussed in the previous sections, the shape functions of membranes do not represent the exact material shape of membranes if there are surface undulations. In this case, the shape functions represent apparent or projected areas, while the undulations are not reflected in the shape functions. In other words, when solving a boundary value problem, calculated shapes are long wavelength projected areas of membranes. Note that when the membrane lateral strain is close to the crossover strain, the calculated shape might be close to the membrane material shape.

### 4.2. Bending Energy Renormalization in the Low-Stain Regime

As depicted in Figure 4a, biological membranes are not smooth in long wavelength scales but show undulations on their surface. Theoretical works had been conducted to account for the effect of these entropic undulations, i.e., excessive areas, in the curvature deformation and to obtain the renormalized bending modulus of coarse-grained lipid membranes in longer wavelength scales [33,34,35,36]. According to previous works, the bending modulus with respect to projected areas (Figure 4a black curve) of a membrane with undulations can be expressed as follows [33,34,35,36]:

(19) $$\kappa_{\mathrm{renormalize}}≅\kappa -\frac{3}{4\pi }k_{B}T\ln\frac{q_{\max}}{q_{\min}}.$$

In Equation (19), the renormalized bending modulus can be determined by the maximum and minimum wavenumber q of membranes.

As similarly conducted for deriving Equation (11), planer membranes parameterized as waves, the curvature energy expressed up to quadratic order, and the equipartition theorem were used to derive the energy functional with respect to the renormalized bending modulus $\kappa_{\mathrm{renormalize}}$ [35,36]. As discussed in ref. [36], the change of the bending modulus with different levels of undulations might not be significant. For example, $\frac{3}{4\pi }k_{B}T\ln\frac{q_{\max}}{q_{\min}}$ is $0.1655\;k_{B}T$, $0.5497\;k_{B}T$, $1.0994\;k_{B}T$, and $1.6491\;k_{B}T$ when $\frac{q_{\max}}{q_{\min}}$ is 2, 10, 100, and 1000, respectively. These values might be small compared to bending moduli which are $10\;k_{B}T$ to $60\;k_{B}T$ (Figure 3).

### 4.3. A Simplified Energy Functional

The energy functional in Equation (18) can be simplified when the strain of membranes is consistent. According to Kim (see Appendix A in supplementary information of ref. [26]), the variation of membrane strain energy, i.e., ${\delta \Psi }_{\mathrm{lateral}}$, can be expressed as follows.

(20) $${\delta \Psi }_{\mathrm{lateral}}=\left(\sigma_{0}\left(\alpha +1\right)\exp\left(c_{1}\alpha \right)+\frac{\sigma_{0}}{c_{1}}\exp\left(c_{1}\alpha \right)-\frac{\sigma_{0}}{c_{1}}\right)\delta A\;\mathrm{for}\;\alpha \leq \alpha_{\mathrm{crossover}},$$

(21) $${\delta \Psi }_{\mathrm{lateral}}=\left(K_{\mathrm{app}}\left(\alpha +1\right)\alpha -K_{\mathrm{app}}\alpha_{\mathrm{cut}\text{-}\mathrm{off}}\;\left(\alpha +1\right)+0.5K_{\mathrm{app}}\alpha^{2}-K_{\mathrm{app}}\alpha_{\mathrm{cut}\text{-}\mathrm{off}}\alpha +\;c_{2}\right)\delta A\;\mathrm{for}\;\alpha >\alpha_{\mathrm{crossover}}.$$

Therefore, when strain α is consistent during mechanical deformation, $\Psi_{\mathrm{total}}$ in Equation (18) can be expressed as follows:

(22) $$\Psi_{\mathrm{total}}=\Psi_{\mathrm{curvature}}+\Psi_{\mathrm{lateral}}\;=\int_{\Omega }^{}\left(2{\kappa H}^{2}+\;\bar{\kappa }\;K\right)\mathrm{dA}+{Τ}_{\alpha }\int_{\Omega }^{}\mathrm{dA},$$

where the constant ${Τ}_{\alpha }$ is

(23) $${Τ}_{\alpha }=\sigma_{0}\left(\alpha +1\right)\exp\left(c_{1}\alpha \right)+\frac{\sigma_{0}}{c_{1}}\exp\left(c_{1}\alpha \right)-\frac{\sigma_{0}}{c_{1}}\;\mathrm{for}\;\alpha \leq \alpha_{\mathrm{crossover}},$$

(24) $${Τ}_{\alpha }=K_{\mathrm{app}}\left(\alpha +1\right)\alpha -K_{\mathrm{app}}\alpha_{\mathrm{cut}\text{-}\mathrm{off}}\left(\alpha +1\right)+0.5K_{\mathrm{app}}\alpha^{2}-K_{\mathrm{app}}\alpha_{\mathrm{cut}\text{-}\mathrm{off}}\;\alpha +c_{2}\;\mathrm{for}\;\alpha >\;\alpha_{\mathrm{crossover}}.$$

The form in Equation (22) is similar to an energy functional employed in many previous works using constant surface tension [2,5,12]. However, ${Τ}_{\alpha }$ is not the tension σ of lipid membranes. Nevertheless, values for σ and ${Τ}_{\alpha }$ are similar to each other as demonstrated in Figure 2a.

In ref. [26], membranes with closed lipid reservoirs were solved by using Equation (22). A predictor–corrector scheme was used for the condition of a fixed total lipid number. The continuum theory was validated in the low-strain regime from experiments by using nanoscale vesicles and magnetic tweezers [26]. Nanomechanical responses measured by modulating the interaction between the vesicles and substrates were reproduced by using the continuum theory. Complex nonlinear mechanical responses, including bistable behaviors observed from the vesicles were directly compared with calculations [26].

## 5. Numerical Methods

Numerical methods to find the equilibrium configuration of lipid membranes described by continuum theories have been investigated in numerous previous works. Different branches of numerical methods are available. First, there are investigations where the Euler–Lagrange equation of the membrane energy functional was derived and solved numerically [8,37]. For example, in a work by Powers et al. [37], ordinary differential equations of the membrane in the rotational symmetric configuration were solved by using relaxation methods to calculate the equilibrium configuration of membrane tethers. In ref. [8], the Euler–Lagrange equation was solved numerically for conical anchor problems as well as the mechanical deformation of vesicles. Secondly, there are numerous works that used finite difference-type schemes [38,39,40]. These works employed triangular surface discretization where values for the differential geometry were calculated. In this paper, the review was focused on numerical methods based on finite element methods (FEMs). For the case of Galerkin FEMs, the validity of numerical results might be supported by the best approximation property of FEMs [41]. In the following paragraphs, several previous methods are introduced.

FEM-style calculations were introduced in works by Feng, Ma, and Klug [42,43]. In the works, the shape of membranes was parameterized by using C^1^-conforming elements. Based on the curvature elasticity theory, the membrane energy was minimized to calculate the equilibrium configuration of membrane vesicles and tethers. Fluid membranes do not have lateral shear elasticity. Therefore, instabilities, i.e., zero-energy modes, can be invoked for nodal motions to the tangential direction of surfaces. In ref. [43], to avoid the distortion of meshes that can be generated due to the zero-energy mode, Hookean springs were considered within finite element meshes. However, the addition of this artificial shear elasticity might need further physical validation for fluid membranes.

Rangarajan and Gao introduced a Galerkin variational method to calculate the equilibrium configuration of lipid membranes [44]. They employed normal offset coordinate systems in deriving variational equations to avoid the zero-energy mode. The shape of lipid membranes was parameterized by using B-spline functions. This nonlinear finite element method used the Newton–Raphson method. Rotational axisymmetric analyses on the adhesion of vesicles, membrane wraps, and membrane tethers were performed. Three-dimensional calculations were also tried in this work [44].

Sauer et al. introduced a method using thin shell elements and the Canham–Helfrich energy functional in modeling lipid membranes [45]. The method used iso-geometric approaches to parameterize the surface of lipid membranes. Quasi-static calculations by using the model were stabilized by adding stiffness components or employing a normal projection method. The model was extended for the calculation of the axis asymmetric membrane [45,46]. However, approaches in modeling thin shells might not be the best to model fluid membranes as they can show unnecessary solid-like behaviors.

Kim introduced an axisymmetric finite element method for lipid membranes [26]. Variational equations were derived for the two-dimension system. However, the motion of nodal degrees of freedom was constrained into the normal direction of reference surfaces to avoid the zero-energy mode. The work also utilized the B-spline function and the Newton–Raphson method to solve nonlinear equations iteratively. Using differential geometry formulas in the Monge patch and parametric derivatives, the derivation of variational equations and the tangential matrix for the Newton–Raphson method is straightforward in this Galerkin FEM [26].

A combined model where the curvature elasticity theory is coupled with a model for the dissipative flow of membranes was introduced [47]. In this work, instead of investigating the variational shape optimization, the time-dependent flow of energies was studied to determine membrane shapes. B-spline functions were used to parameterize the rotational axisymmetric membrane configuration and the Galerkin method was used for the equation system. The approaches presented in this work were extended for the three-dimensional mechanical relaxation of vesicles [48].

## 6. Biological Applications

The curvature elasticity theory and surface tension of lipid membranes were used in various biological problems alone or in combination with other biophysical equations. Those theoretical investigations, together with cumulated biological data on cell membranes, have provided invaluable insight into various cellular physiologies, including membrane remodeling, exo- and endocytosis, the deformation of nuclear pores, the activation of ion channels, and membrane receptor-mediated mechanotransduction. In the following paragraphs, previous works on these cellular processes using the continuum theories were reviewed.

Calculations for membrane remodeling were performed for the cell surface membrane as well as the membrane of cellular organelles. For example, Atilgan et al. investigated the protrusion of membranes driven by dynamic actin filaments [2]. Membrane energies defined by the mean curvature and surface tension were used to predict how the number and arrangement of actin filaments, the thermal fluctuation of membranes, and the tethering of membranes to cytoskeletons affect the protrusion dynamics [2]. Mechanical deformation of membranes was investigated for cellular organelles as well. For example, a membrane squeezing problem was addressed by using a combined equation for membrane curvatures, surface tension fields, and a volume constraint to investigate the fission of mitochondria [3]. According to the work, membrane proteins and conical lipids can together trigger buckling instability and generate an extreme fission neck radius. In addition, by combining the curvature energy and ultra-low membrane tension, and using the surface evolver program [4], the nanostructure of the endoplasmic reticulum was calculated [5]. Mechanical responses of nuclear pore-like structures were also investigated by using the continuum membrane theories [6,7].

The fusing and fission of membrane vesicles are crucial in the exo- and endocytosis processes, respectively. By introducing the membrane curvature energies, the surface tension fields, and the volume constraint as similarly conducted in previous works [3,8], membrane invagination shapes were calculated to investigate the growth of vesicles driven by actins [49]. In a work by Irajizad et al., membrane remodeling driven by clathrin proteins was investigated by using the Canham–Helfrich theory [9].

The interaction between mechanosensitive (MS) ion channels and lipid bilayers had been investigated in numerous previous works by using the continuum theories. For example, Wiggins and Philips investigated the interaction between lipid bilayers and the mechanosensitive channel of large conductance (MscL). They calculated free energies generated by the lipid–channel interaction that is on the same order of measurements [11]. The membrane resultant calculated by using the Canham–Helfrich model was used to investigate the activation of hair cell MS channels [12]. Similarly, membrane free energies at the tip of stereocilia calculated by using the curvature elasticity and surface tension were able to activate hair cell MS channels [13]. More recently, the curvature elasticity theory reproduced membrane shapes around Piezo channels inserted in vesicles [14]. In addition, the theory predicted responses for the activation of Piezo channels [15].

Mechanical deformation of membranes was also investigated for responses mediated by cell surface receptors. Mechanical switches in cell membranes that modulate hair-cell and integrin-mediated mechanotransduction processes were identified from membrane calculations in the low-strain regime [13,16]. In addition, nanomechanical responses measured by pulling the membrane-proximal ectodomain of cadherins in living cell surfaces were directly analyzed by using the continuum theory in the low-strain regime [26].

## 7. Conclusions

The curvature elasticity theory originally introduced by Canham [17] and Helfrich [18] and the constitutive relation between surface tension and strain investigated by Evans and his colleagues [19,20] have been central for many mechanobiological problems. In this paper, previous works on theoretical formulations, numerical methods, and biological applications of the continuum theories for the mechanical deformation of lipid membranes were reviewed.

Despite many successful applications of the theories and methodologies in describing biological membranes, the following topics can be further investigated as future works to improve current approaches. First, continuum theories that can describe details of real cell membranes, such as lipid sorting, lateral strain gradients, and mobile transmembrane and crosslinker proteins can be further investigated. Second, theoretical methods to fully couple molecular models into continuum frameworks can be developed. This might be particularly important as protein structural models can be simulated within the continuum model frameworks. The development of fully coupled and realistic membrane-cytoskeleton models is also important. Similarly, the development of continuum models for the full nuclear envelope and the endoplasmic reticulum with greater molecular and nanomechanical detail might be also crucial. Together with super resolution, nanoscale, and single-molecule experimental methods, these theoretical improvements can stimulate the limit of biological membrane sciences and technologies.

## Data Availability

No new data were created or analyzed in this study. Data sharing is not applicable to this article.
